# The Reactome pathway Knowledgebase

**DOI:** 10.1093/nar/gkv1351

**Published:** 2015-12-09

**Authors:** Antonio Fabregat, Konstantinos Sidiropoulos, Phani Garapati, Marc Gillespie, Kerstin Hausmann, Robin Haw, Bijay Jassal, Steven Jupe, Florian Korninger, Sheldon McKay, Lisa Matthews, Bruce May, Marija Milacic, Karen Rothfels, Veronica Shamovsky, Marissa Webber, Joel Weiser, Mark Williams, Guanming Wu, Lincoln Stein, Henning Hermjakob, Peter D'Eustachio

**Affiliations:** 1 European Bioinformatics Institute (EMBL-EBI), European Molecular Biology Laboratory, Wellcome Trust Genome Campus, Hinxton, Cambridge CB10 1SD, UK; 2 Ontario Institute for Cancer Research, Toronto, ON M5G0A3, Canada; 3 College of Pharmacy and Health Sciences, St John's University, Queens, NY 11439, USA; 4 NYU School of Medicine, New York, NY 10016, USA; 5 Cold Spring Harbor Laboratory, Cold Spring Harbor, NY 11724, USA; 6 Department of Molecular Genetics, University of Toronto, Toronto, ON M5S 1A1, Canada; 7 National Center for Protein Sciences, Beijing, China

## Abstract

The Reactome Knowledgebase (www.reactome.org) provides molecular details of signal transduction, transport, DNA replication, metabolism and other cellular processes as an ordered network of molecular transformations—an extended version of a classic metabolic map, in a single consistent data model. Reactome functions both as an archive of biological processes and as a tool for discovering unexpected functional relationships in data such as gene expression pattern surveys or somatic mutation catalogues from tumour cells. Over the last two years we redeveloped major components of the Reactome web interface to improve usability, responsiveness and data visualization. A new pathway diagram viewer provides a faster, clearer interface and smooth zooming from the entire reaction network to the details of individual reactions. Tool performance for analysis of user datasets has been substantially improved, now generating detailed results for genome-wide expression datasets within seconds. The analysis module can now be accessed through a RESTFul interface, facilitating its inclusion in third party applications. A new overview module allows the visualization of analysis results on a genome-wide Reactome pathway hierarchy using a single screen page. The search interface now provides auto-completion as well as a faceted search to narrow result lists efficiently.

## INTRODUCTION

At the cellular level, life is a network of molecular reactions that include signal transduction, transport, DNA replication, protein synthesis and intermediary metabolism. In Reactome, these processes are systematically described in molecular detail to generate an ordered network of molecular transformations, resulting in an extended version of a classic metabolic map described by a single, consistent data model (1). The Reactome Knowledgebase thus systematically links human proteins to their molecular functions, providing a resource that functions both as an archive of biological processes and as a tool for discovering unexpected functional relationships in data such as gene expression pattern surveys or somatic mutation catalogues from tumour cells.

Since its inception 12 years ago, Reactome has grown to include (version 54—September 2015) entries for 8701 human genes (43% of the 20 296 predicted human protein-coding genes—http://Jul2015.archive.ensembl.org/Homo_sapiens/Info/Annotation), supporting the annotation of 18 658 specific forms of proteins distinguished by co- and post-translational modifications and subcellular localizations. These entities function together with 1540 small molecules as substrates, catalysts and regulators in 8770 reactions annotated on the basis of data from 20 708 literature references. These tallies include 1155 mutant variants and their post-translationally modified forms derived from 249 gene products, used to annotate 787 disease-specific reactions, tagged with 262 Disease Ontology terms (2). Recent additions include hedgehog signalling, host cell damage by bacterial toxins and extended annotations of DNA repair processes.

Here, we focus on three aspects of Reactome that have been extensively redesigned and improved since its last review in NAR (1): the web visualization and navigation browser, the toolkit for data analysis and the search utility.

## PATHWAY OVERVIEW

Pathways in Reactome are organized hierarchically, grouping detailed pathways for translation, protein folding and post-translational modification into larger domains of biological function like protein metabolism. This hierarchical organization largely follows that of the Gene Ontology (GO) biological process hierarchy (3,4). Reactome thus implements a pathway graph.

**Figure 1.**
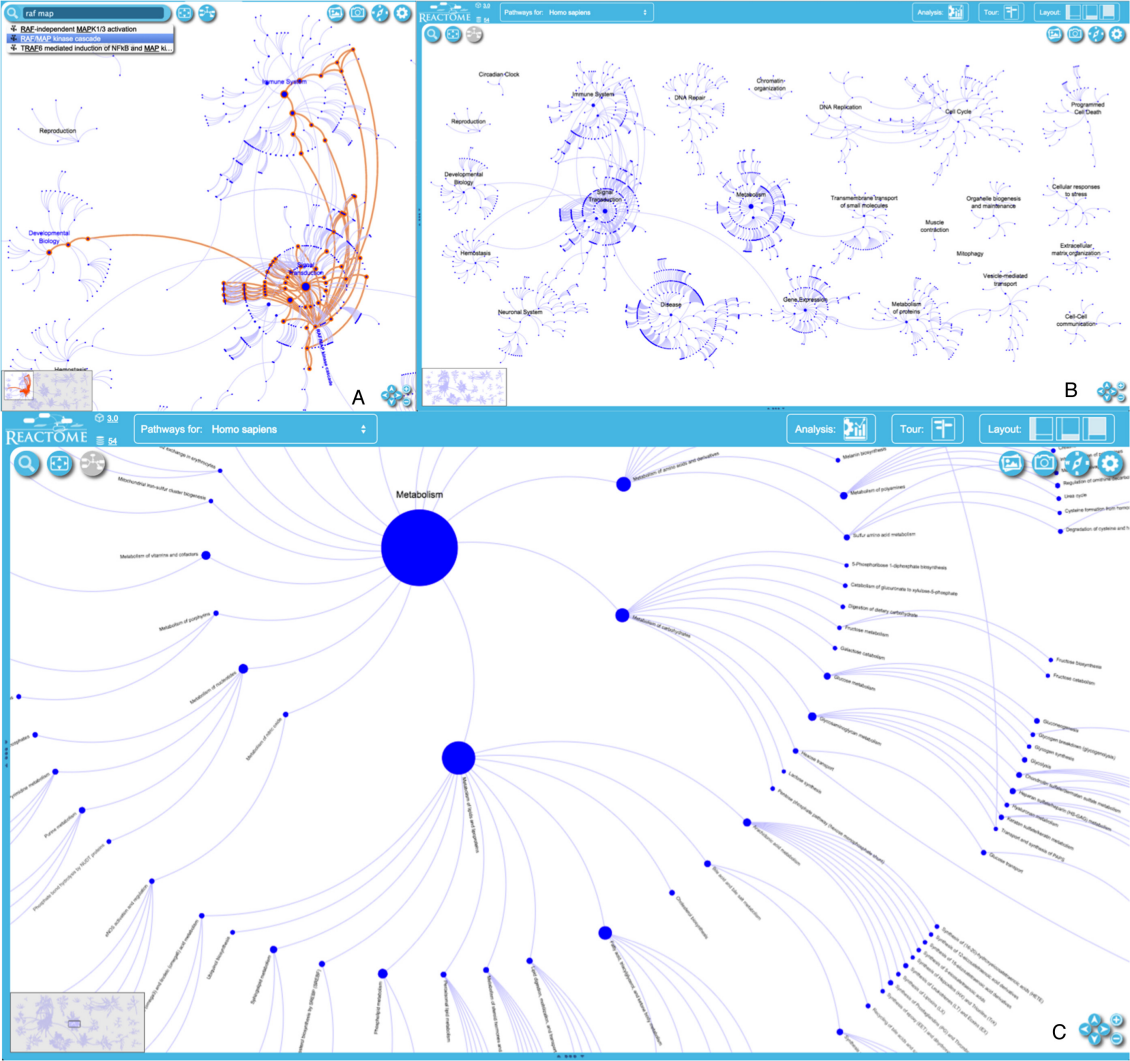
Pathway Overview. The entire pathways overview map (**A**). The RAF/MAP kinase cascade pathway is highlighted to show its involvement in multiple bursts (**B**). A zoomed-in view of the Metabolism burst showing individual subpathway groups (**C**).

The pathway overview visualization provides an overview of all Reactome pathways, that highlights parent–child relationships and processes that are shared between pathways (Figure 1; http://www.reactome.org/PathwayBrowser/). In this view the 24 major Reactome pathway groups are each organized as a roughly circular ‘burst’. The central node of each burst corresponds to the uppermost level of the Reactome event hierarchy (e.g. hemostasis, gene expression, signal transduction). Concentric rings of nodes around the central node represent successive more specific levels of the event hierarchy (e.g. signal transduction → signalling by FGFR → signalling by FGFR1). The arcs connecting nodes between successive rings within a burst represent parent–child (is-a) relationships in the event hierarchy. When a specific pathway like RAF/MAP kinase cascade is shared by more than one burst, arcs connect its nodes between bursts. A node's size is proportional to the number of physical entities (proteins, complexes, chemicals) it contains. Bursts are manually positioned to minimize crossing of arcs between bursts, and new bursts are manually added to the layout. With each new data release, a layout algorithm automatically adjusts the locations of existing nodes within the bursts to accommodate newly added nodes, maintaining spacing within rings and avoiding overlaps of nodes from neighbouring bursts, while minimizing displacement of the groups from their previous positions in the overview. Changes in the overall organization of the whole reaction network due to updates are thereby minimized, helping users identify and track areas of interest. This layout provides a legible, stable, informative overview and entry point to Reactome content even as the number of annotated proteins and processes in Reactome continues to increase.

## DIAGRAM VIEWER

The new version of the diagram viewer reduces the loading time for diagrams and data, as well as the analysis results displayed on top of them. It provides visual feedback for common actions like hovering and focusing, has smoother transitions for zooming and selection and implements a mechanism to coordinate the amount of detail shown with the zoom level—as the user zooms into specific parts of a diagram, more detailed information is progressively overlaid. A new search tool enables users to find items of interest within a diagram.

**Figure 2.**
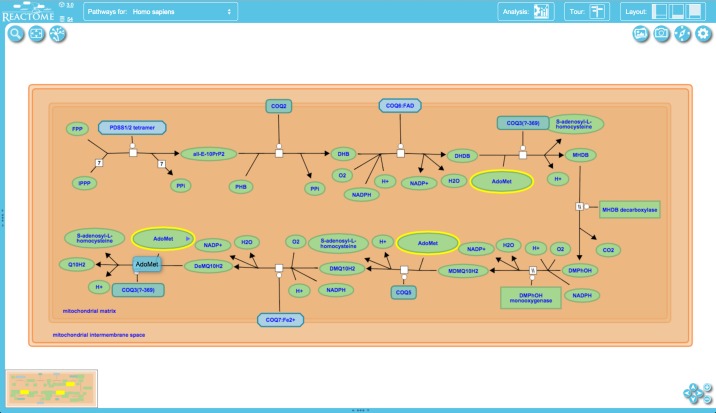
Diagram viewer. The central panel shows details of reactions and participating molecules in the nine-step process of ubiquinol (ubiquinol-10, Q10H2) biosynthesis. Buttons around the panel support functions including panning and zooming (lower right), changing the view (upper left) and downloading a snapshot of the pathway (upper right).

To support efficient navigation and searching within diagrams we have implemented a directed graph data structure which holds information such as the identities of the physical entities that make up complexes or sets and annotated preceding/following relationships between reactions in a pathway. This data structure is linked to the entities and events displayed in the diagram and takes advantage of graph traversing algorithms to support features such as rapid drilling down into complexes to reveal their components and navigation to all occurrences of an entity, both as an individual entity or as part of a larger composite entity, when present multiple times in a diagram (e.g. pyrophosphate (PPi) and H^+^ in Figure 2).

## PATHWAY BROWSER

**Figure 3.**
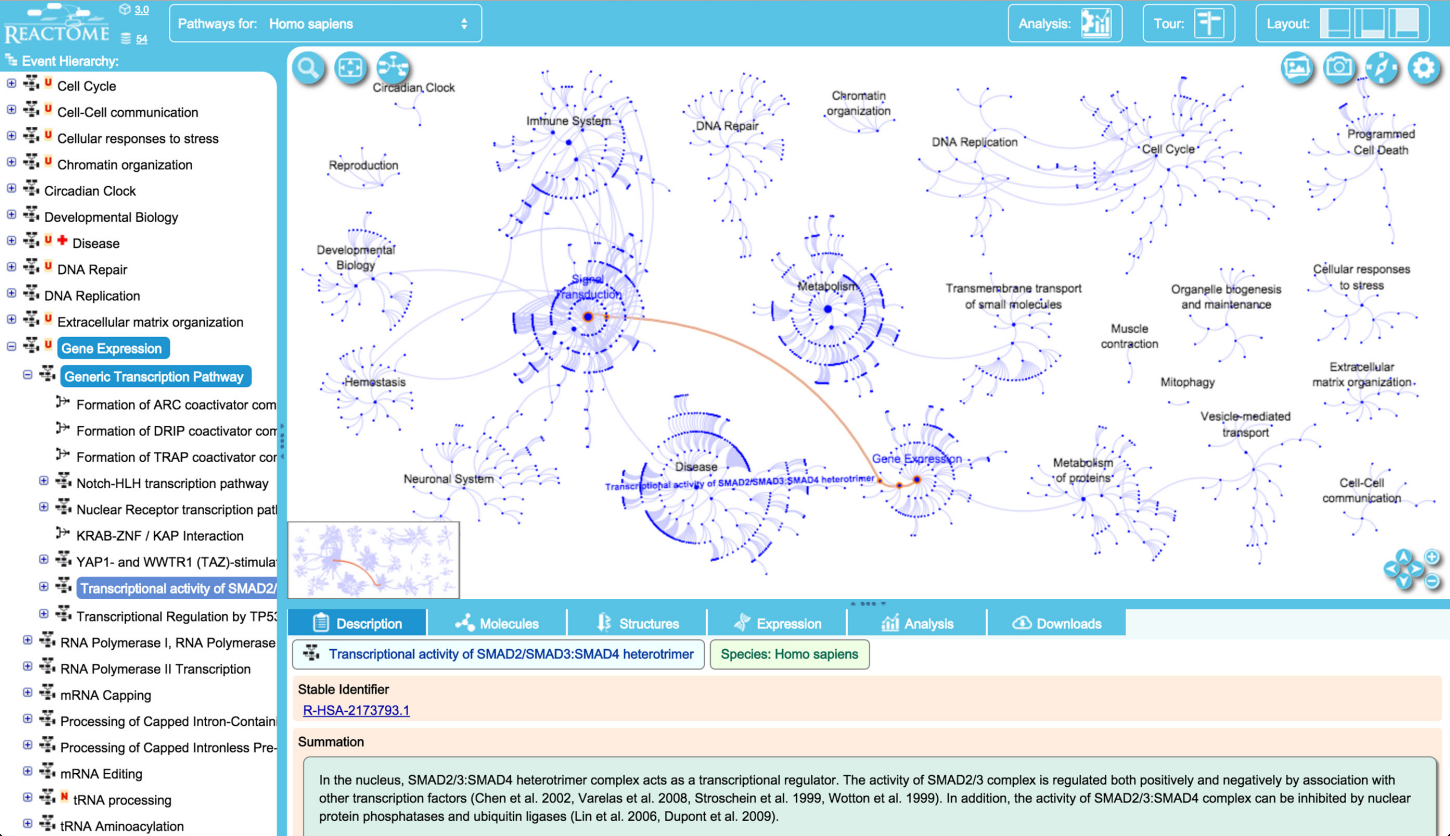
Pathway browser view centred on the ‘gene expression’ top-level pathway. Access to subpathways is provided via the hierarchical display of events on the left and by clicking on event nodes in the pathway display (viewport). Details for the selected event are shown in the panel under the pathway display. Buttons at the right of the top bar show the current version of our software (3.0) with access to our Github software repository, and the current version of our data (release 54). A button in the top bar provide access to the analysis tools (see below, Figure 4). Clicking on the layout buttons closes and re-opens the hierarchical display and details panels. The ‘tour’ button provides access to a brief video tour of the main features of the web site. Clicking on the gearwheel icon in the upper right corner of the pathway diagram provides access to a tool to customize diagram colouring and to an ‘About …’ pop-up that briefly describes pathway diagram features and contains a link to the detailed users’ guide. (This guide is also accessible via the ‘documentation’ drop-down menu at the top of the home page).

The pathway browser (http://www.reactome.org/PathwayBrowser/) (Figure 3) has been updated to reduce its loading time and provide a more attractive user interface. Buttons for widely used actions have been made more prominent, icons and colour schemes have been re-designed, and features including colour profiles can be customized by users. The pathway browser opens with the ‘starburst’ overview explained in the previous section. This overview is integrated with a diagram viewer that shows molecular details of pathways and individual reactions. When the pathway browser is loaded, the events hierarchy and the details panel appear on the left and bottom of the viewport, respectively. The pathways overview widget is placed in the main viewport. Double clicking a pathway in the events hierarchy or its node in the main viewport will trigger a smooth, animated zoom in the main viewport to reveal the diagram for the pathway.

All display components are tightly connected, so that actions in one component will cause updates in others to consistently present information across the different display elements in accordance with the user's selection. For example, choosing a reaction node or a physical entity glyph in the pathway diagram will trigger an update of the information displayed in the details panel under the pathway diagram and the events hierarchy panel on the left.

## PATHWAY ANALYSIS

Reactome's annotated data are a part of list that shows what could happen if all annotated proteins and small molecules were present and active simultaneously in a cell. By overlaying an experimental dataset on these annotations, such as a list of genes activated in response to an experimental stimulus or expressed in transformed cells but not their normal counterparts, a user can search for patterns in the dataset such as modulation of specific pathways. By overlaying quantitative expression data or time series, a user can visualize the extent of change in affected pathways and its progression.

Changing use patterns and growing data content are rapidly increasing performance demands for Reactome Pathway Analysis; high-throughput datasets often contain thousands or tens of thousands of identifiers. To address this challenge, we have re-implemented the analysis system, which now achieves interactive speed for genome-wide datasets, typically providing results for a dataset with 20 000 identifiers in less than 3 s. In addition to high execution speed, we now offer fine-grained results across all pathway levels in the Reactome events hierarchy. We provide a measure of target pathway coverage not only in terms of identified molecules, but also in terms of hit reactions per pathway.

**Figure 4.**
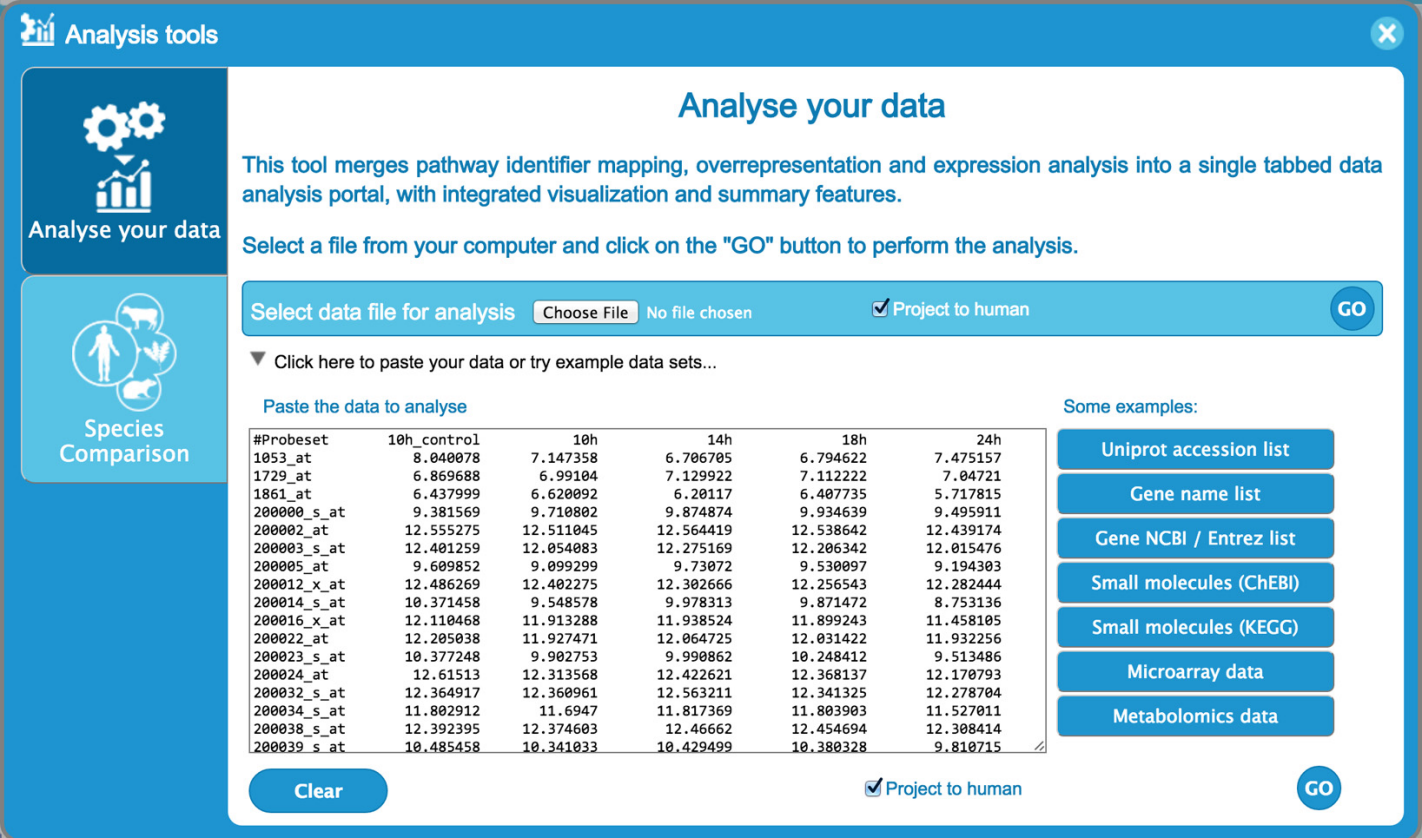
Analysis tool user data submission interface, showing time-series data. Each row represents data for a different gene. Columns contain an identifier (probe set, gene name, etc.) on the left and expression values for four time points to the right, entered as tab-delimited text. UniProt identifiers, gene names and Affimetrix identifiers, among others, can be submitted. The ‘project to human’ box at the bottom of the form, which is selected by default, causes any non-human identifiers in the data to be replaced by their human equivalents and the latter to be used for the analysis. Instructions for formatting data and lists of acceptable identifiers are provided in the users’ guide (Figure 3).

The pathway analysis data submission interface is launched by selecting the analysis button located in the right top corner of the pathway browser. Once the user data is submitted by uploading or pasting a file into the allocated text area (Figure 4), the analysis is performed on the server side with the results shown in the pathway browser.

**Figure 5.**
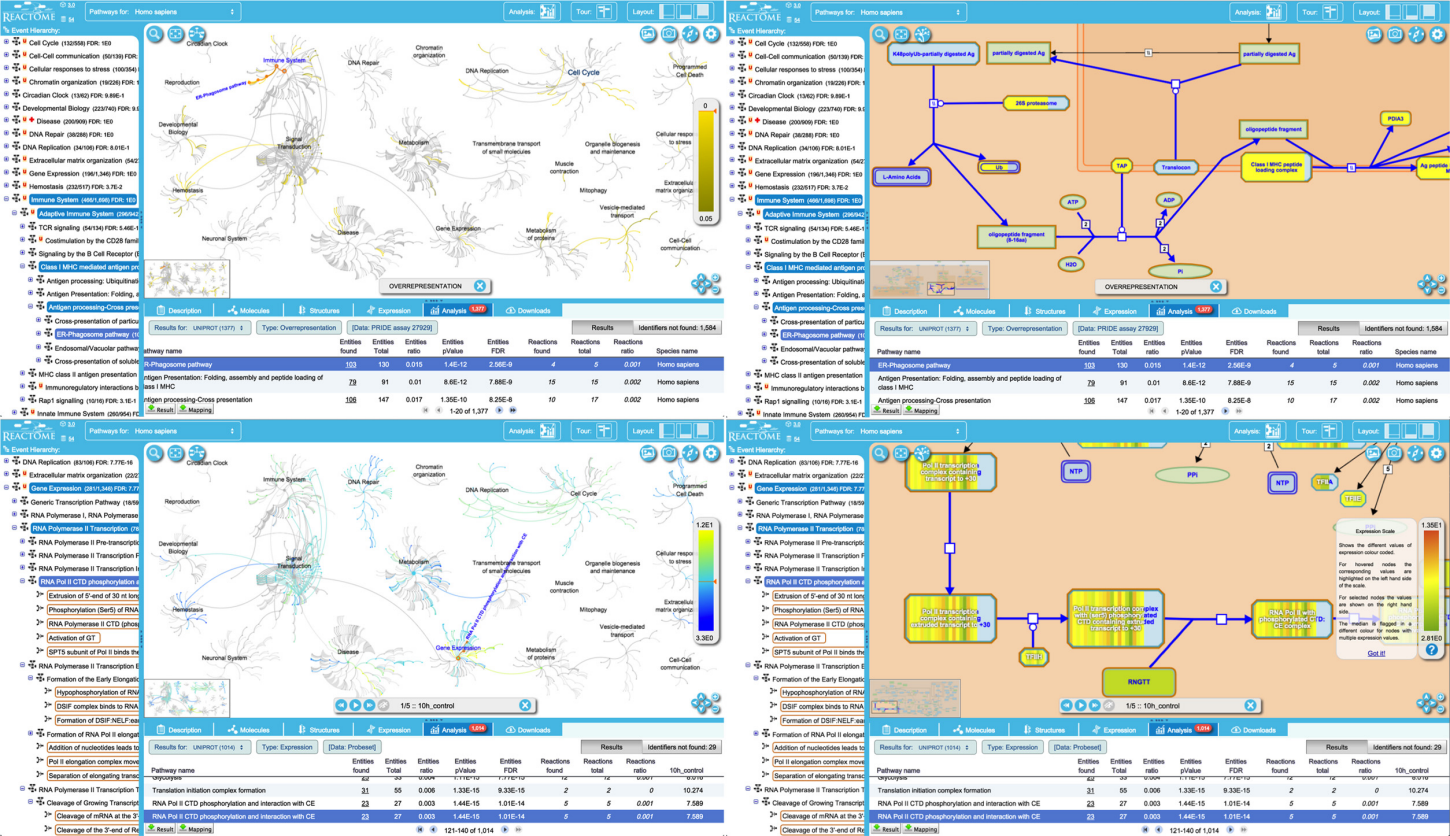
Analysis results. Top panels, an analysis of a PRIDE dataset (assay 27 929—http://www.ebi.ac.uk/pride/ws/archive/protein/list/assay/27929.acc in project PXD000072—http://www.ebi.ac.uk/pride/archive/projects/PXD000072) to identify proteins over-expressed in activated human platelet releasate (5). Bottom panels, an expression analysis. Left panels show overlays on the pathways overview; right panels are an overlay of the data for a selected pathway on the pathway diagram. The details panel at the bottom lists results and statistics for each pathway, including numbers of identifiers in the submitted dataset that did not match anything in the Reactome dataset. A binomial test is used to calculate the probability shown for each result, and the *P*-values are corrected for the multiple testing (Benjamini–Hochberg procedure) that arises from evaluating the submitted list of identifiers against every pathway.

A new details panel displays results in tabular form. We have taken advantage of the new Reactome pathway overview visualization to show the analysis results as an overlay, allowing users to start with a high-level overview of results and then zoom in on areas of interest. Selecting a row in the results table highlights the corresponding events in the hierarchy and focuses the pathway overview on the corresponding burst, or loads the corresponding pathway diagram (Figure 5).

Analysis results are temporarily stored on the Reactome server. The storage period depends on usage of the service but is at least 7 days. Stored results are available via the token assigned to the results file when it is created and displayed in the URL for the results report. The token can be shared and allows later access through the API.

High-throughput pathway analysis is supported by a new RESTFul web service interface (API), documented in detail (http://www.reactome.org/AnalysisService/), which allows use of the Reactome server for batch dataset analysis. Over-representation and expression data analysis can be performed against the Reactome database (***/identifier*** and ***/identifiers*** methods) as well as species comparison (***/species*** method). Once the data analysis or species comparison has been performed, a *token* is included in the client results allowing further service calls to refine the initial findings (***/token*** and ***/download*** methods).

## FULL-TEXT SEARCH

**Figure 6.**
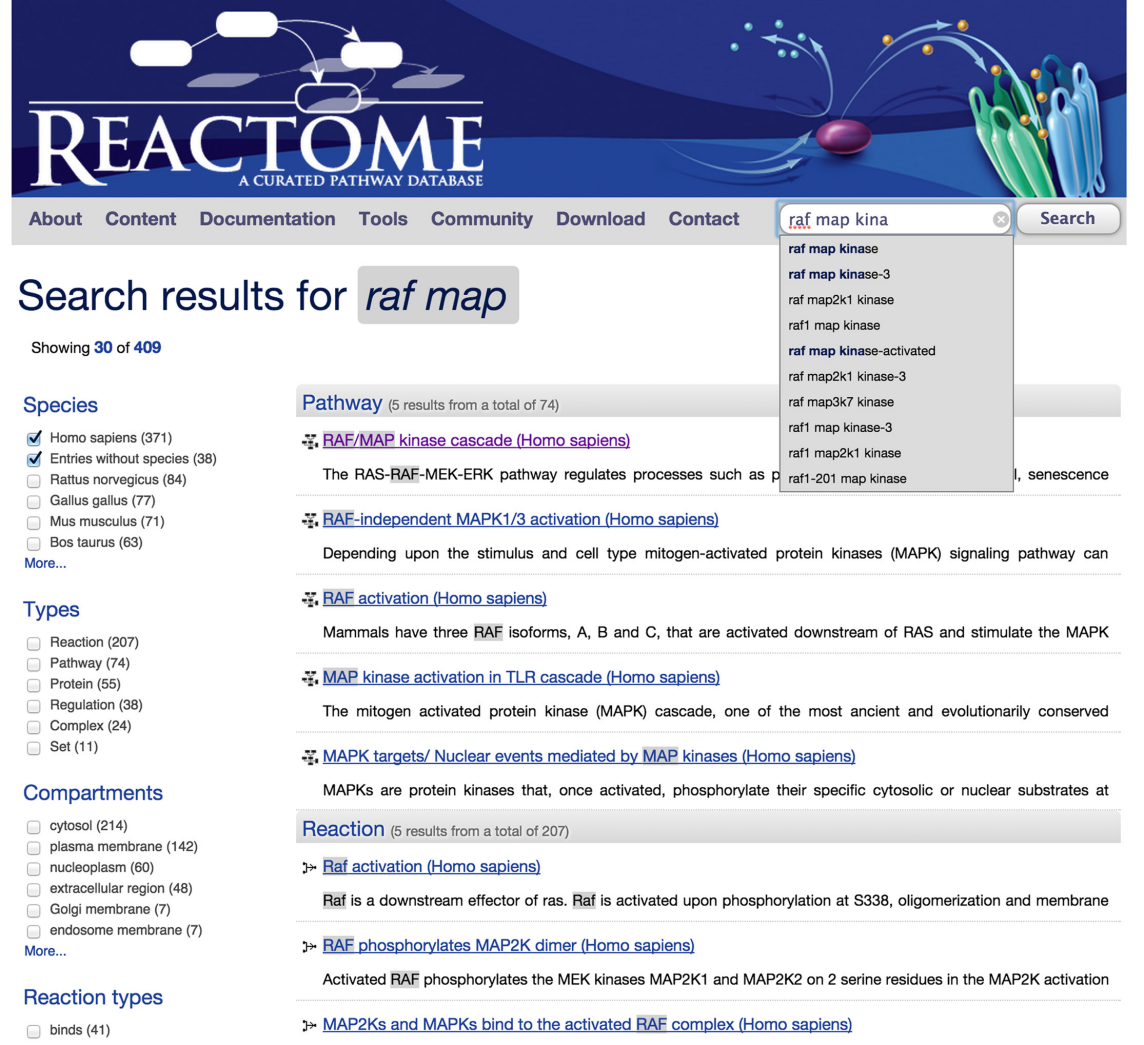
Redesigned search interface, showing term auto suggestion, grouping of results and highlighting of search terms in the results. The check boxes along the left side of the results page allow results to be further limited by species, data type, subcellular location and other parameters.

The search tool has been redesigned to provide fast data access and incorporate additional data type attributes, yielding more accurate search results (Figure 6). The search core employs Solr, a high performance scalable full-text search engine specifically designed to search through large datasets. New features include filtering, results grouping, hit highlighting, spell checking and auto completion as the user types terms into the search text box.

## CONCLUSIONS

The changes to the Reactome site and data analysis tools described here provide users with faster, easier access to Reactome data increasing its utility both as an archive of known human biology and as a tool for generating and testing experimental hypotheses. The newly developed tools scale well to support the continued growth of Reactome content and its extension to new data types such as non-coding RNAs. These tools have been designed to support persistent growth in the number, size and complexity of user-supplied datasets for analysis.
